# Evaluation of Genomic Prediction for Fusarium Head Blight Resistance with a Multi-Parental Population

**DOI:** 10.3390/biology10080756

**Published:** 2021-08-06

**Authors:** Wentao Zhang, Kerry Boyle, Anita Brule-Babel, George Fedak, Peng Gao, Zeinab Robleh Djama, Brittany Polley, Richard Cuthbert, Harpinder Randhawa, Robert Graf, Fengying Jiang, Francois Eudes, Pierre R. Fobert

**Affiliations:** 1 Aquatic and Crop Resources Development, National Research Council of Canada, Saskatoon, SK S7N 0W9, Canada; Kerry.Boyle@nrc-cnrc.gc.ca (K.B.); Peng.Gao@nrc-cnrc.gc.ca (P.G.); Brittany.Polley@nrc-cnrc.gc.ca (B.P.); 2 Department of Plant Science, Agriculture Building, University of Manitoba, Winnipeg, MB R3T 2N2, Canada; Anita.Brule-Babel@umanitoba.ca; 3 Ottawa Research and Development Centre, Agriculture and Agri-Food Canada, Ottawa, ON K1A 0C6, Canada; George.Fedak@AGR.GC.CA (G.F.); Zeinab.RoblehDjama@AGR.GC.CA (Z.R.D.); 4 Swift Current Research and Development Centre, Agriculture and Agri-Food Canada, Swift Current, SK S9H 3X2, Canada; Richard.Cuthbert@AGR.GC.CA; 5 Lethbridge Research and Development Centre, Agriculture and Agri-Food Canada, Lethbridge, AB T1J 4B1, Canada; Harpinder.Randhawa@AGR.GC.CA (H.R.); robert.graf@AGR.GC.CA (R.G.); Fengying.Jiang@AGR.GC.CA (F.J.); francois.eudes@AGR.GC.CA (F.E.); 6 Aquatic and Crop Resources Development, National Research Council of Canada, Ottawa, ON K1A 0R6, Canada

**Keywords:** Fusarium head blight, prediction accuracy, genomic selection, model

## Abstract

**Simple Summary:**

Genomic selection is a promising approach to select superior wheat lines with better resistance to Fusarium head blight. The accuracy of genomic selection is determined by many factors. In this study, we found a training population with large size, genomic selection models incorporating biological information, and multi-environment modelling led to considerably better predictabilities. A training population designed by the coefficient of determination (CDmean) could increase accuracy of prediction. Relatedness between training population (TP) and testing population is the key for accuracies of genomic selection across populations.

**Abstract:**

Fusarium head blight (FHB) resistance is quantitatively inherited, controlled by multiple minor effect genes, and highly affected by the interaction of genotype and environment. This makes genomic selection (GS) that uses genome-wide molecular marker data to predict the genetic breeding value as a promising approach to select superior lines with better resistance. However, various factors can affect accuracies of GS and better understanding how these factors affect GS accuracies could ensure the success of applying GS to improve FHB resistance in wheat. In this study, we performed a comprehensive evaluation of factors that affect GS accuracies with a multi-parental population designed for FHB resistance. We found larger sample sizes could get better accuracies. Training population designed by CDmean based optimization algorithms significantly increased accuracies than random sampling approach, while mean of predictor error variance (PEVmean) had the poorest performance. Different genomic selection models performed similarly for accuracies. Including prior known large effect quantitative trait loci (QTL) as fixed effect into the GS model considerably improved the predictability. Multi-traits models had almost no effects, while the multi-environment model outperformed the single environment model for prediction across different environments. By comparing within and across family prediction, better accuracies were obtained with the training population more closely related to the testing population. However, achieving good accuracies for GS prediction across populations is still a challenging issue for GS application.

## 1. Introduction

Fusarium head blight (FHB), or scab, mainly caused by the fungus *Fusarium graminearum* Schwabe [*telomorph: Gibberella zeae Schw.* (Petch)], is becoming the most destructive fungal disease for wheat production in both Canada and USA [1]. Fusarium infected grains display shrunken shapes (Fusarium damaged kernels [FDK]) and appear white or pink in color (bleaching) [2]. This results in severe yield and quality losses for wheat crops [1,3]. In addition, grains infected by the *F. graminearum* pathogen also accumulate the mycotoxin deoxynivalenol (DON), which represents a severe food and feed safety concern [1]. Annual losses attributed to FHB in Canada are in the hundreds of millions of dollars [4]. While in the USA, during the 2015–2016 season, it was estimated that FHB caused an economic loss of US $4.2 billion [5]. 

Integration of agronomic practices and chemical fungicides with genetic resistance is the most effective means to manage FHB [6,7]. Due to its lower cost, higher efficacy, and environmental benefit, genetic resistance is favored [7]. The genetic architecture underlying FHB resistance is quantitative in nature, controlled by multiple minor genes. To date, more than 500 quantitative trait loci (QTL) for FHB resistance on all 21 wheat chromosomes have been identified [8,9,10]. In addition, the genetics of FHB resistance contains multiple components, which can mainly be defined as four types. These include: (1) resistance to initial infection (Type I, incidence (INC)); resistance to fungal spread across the wheat head (Type II, severity (SEV)); resistance to kernel infection (Type III, Fusarium damaged kernels (FDK)); (2) tolerance to infection, and (3) accumulation of deoxynivalenol (DON) toxin (Type IV) [2,11,12,13,14]. Previous studies indicated that the genetic architectures of these different components are either only partially shared, or independent [12,15,16,17]. In addition to physiological (active) resistance mechanisms, developmental related traits like flowering time, plant height, and spike morphological traits also affect FHB resistance levels through disease escape (passive) mechanisms [12,14,18,19,20,21,22,23,24,25,26]. For example, a negative association between plant height and FHB incidence and severity was observed in numerous studies [19,20,23,25]. Finally, genotype by environmental interactions (GEI) also significantly affect FHB resistance [2,21,26], thus a lower to moderate heritability has been observed for FHB resistance traits [27]. These represent challenges in selecting for FHB resistance in the field using visual selection. 

The nature of FHB resistance traits also represents challenges in the application of the QTL approach, which relies on sufficient statistical power of the mapping populations to improve resistance. Genomics selection/prediction (GS) that can address these limitations, is therefore an ideal approach to improve FHB resistance [14,28,29,30,31]. The strength of GS for FHB resistance is that it uses genome-wide marker information to calculate genomic estimated breeding values (GEBV) and makes selection based on GEBVs instead of phenotypes [32,33]. In addition, GS can increase genetic gain and accelerate selection in the breeding program [32,33]. Recently, the advent of affordable high-throughput genotyping approaches, such as SNP arrays and genotyping-by-sequencing, has allowed GS to be a feasible practice for predicting complex traits in breeding programs. 

The success of applying GS for complex traits is mainly determined by the prediction accuracy/precision (PA). A broad range of factors affect the PA of GS. As reviewed by Voss-Fels et al. [34] and Xu et al. [35], key factors may include, but are not limited to, trait heritability, marker density, population size and structure, relatedness between the TP and the testing population, and statistical models. In general, the PA was found to increase along with the increase of heritability for the target trait [36]. Depending on the property of the population, high density markers were required for the natural populations, while moderate density markers were needed for biparental populations [32,37,38]. Population size was also critical for the accuracy of the GS. Batternffield et al. [39] showed that when a population size increased from 250 to 4095 lines, PA for wheat quality traits increased gradually. A similar pattern was also observed in soybean when TP size reached 4000 lines [40]. However, diminishing returns were observed beyond *N* = 200 for FHB resistance traits in barley [28], *N* = 192 for wheat FHB resistance traits [30] and *N* = 150 in rice for flower time and plant height traits [41]. In general, the size of the TP required depends on the genetic relatedness between the TP and testing population. A smaller TP with a good PA can be achieved when the TP and testing population are closely related [42,43]. In addition, by applying optimization algorithms to design the TP, better accuracies can be achieved [41,44,45,46,47]. 

A broad range of statistical models can be implemented in GS to predict the complex traits, as reviewed by Xu et al. [35]. These models varied in assumptions with different parameters and showed varied accuracies on a trait-by-trait basis [48,49,50]. Models including ridge regression best linear unbiased prediction (RR-BLUP; equal to Genomic best linear unbiased prediction (GBLUP)) and Bayesian’s models [51] have been predominantly applied in GS. Recently, these models were further advanced from single environment models to multi-trait and multi-environment models by incorporating the modeling of GEI [52,53], allowing better predictability of GS across different environments. In addition, research findings with both simulated and empirical datasets have asserted that PA can be considerably improved by incorporating prior known large-effect QTL identified by association mapping or linkage mapping [54,55,56].

GS has been evaluated to predict FHB resistance traits in a number of previous reports [29,30,31,57,58]. However, only a limited number of factors contributing to accuracies of GS for FHB resistance traits were investigated in these studies. Thus, a better understanding of the precision of GS on FHB resistance is needed. In the present study, we aimed to identify and characterize factors that affect accuracies of GS for FHB resistance by analysis of a multi-parental population that we developed previously [17]. These factors include sample size, GS models, TP optimization, the incorporation of prior QTL, multi-trait and multi-environment models and within family and across family predictions. 

## 2. Materials and Methods

### 2.1. Plant Materials

A multi-parental spring wheat (*Triticum aestivum* L.) population (double haploid population) derived from the cross of FL62R1 (as common parental line) with Stettler, Muchmore, and Emerson was developed as described previously [17]. FL62R1 is an Eastern Canadian spring wheat line with comparable resistance to Sumai 3, developed by Comeau et al. [59] with a systemic breeding approach. Stettler and Muchmore are two semi-dwarf, high-yielding Canada Western Red Spring (CWRS) wheat cultivars [60,61] and Emerson is Canada Western Red Winter (CWRW) variety that is the first Canadian wheat to register as an R rating for FHB resistance [62]. The Stettler population (FS) contained 161 lines and the Muchmore (FM) population consisted of 201 DH lines. The Emerson (FE) population has 218 spring type DH lines. 

### 2.2. Disease Inoculation and Phenotyping

The multi-parent population was evaluated for FHB resistance in disease nurseries at Carman, Manitoba (MB) and Ottawa, Ontario (ON) in 2015 and 2016 with three biological replications and a random complete block design (RCBD) in single meter rows. Disease trait evaluation and phenotyping were described in detail by Zhang et al. [17]. The evaluated FHB and related traits included INC, SEV, FDK, and DON levels. Days to anthesis (DA) and plant height (HT) were also recorded, excluding in 2015 at Carman, MB, where no HT data was available. 

### 2.3. Genotyping

The multi-parental population was genotyped with the Illumina iSelect 90K SNP array [63] and raw data were processed and cleaned as described by Wang et al. [63] using the diploid version of GenomeStudio (Illumina, San Diego, CA, USA). High quality SNP markers from GenomeStudio were further cleaned with the R/QTL package and redundant markers were removed. Finally, a high-quality marker dataset with 4205 informative SNP markers was generated for the GS analysis. 

### 2.4. Evaluation of GS

#### 2.4.1. Prediction Models

Seven genomics selection (GS) models, including BayesA, BayesB, BayesC, and Bayesian least absolute shrinkage and selection operator (BLASSO), Bayesian Ridge Regression (BRR), reproducing kernel Hilbert space (RKHS) and RR-BLUP were evaluated by R packages BGLR or rrBLUP [52,64]. Marker effects were estimated by Y = Xβ + Zμ + ε, where Y is the vector of phenotypes, β is the vector of fixed effects, μ is the vector of random marker effect, X and Z are design matrices. ε is the vector of residual error, which assumed a normal distribution as var(ε) ~N (0, Iδ^2^_ε_). I is defined as the identity matrix and δ^2^_ε_ is the residual variance. When QTL were incorporated as fixed effects, the peak markers identified from Zhang et al. [17] were passed to X vector, otherwise, vector of 1 was used. 

#### 2.4.2. Optimization TP

Two optimization algorithms, mean of the coefficient of determination (CDmean) and mean of predictor error variance (PEVmean) proposed by Rincent et al. [46] were implemented in R the package TrainSel [65] to design the training population. A randomly sampled training population was also applied and compared with these optimized training populations generated by CDmean and PEVmean. Training populations were set with sizes 50 and 100 lines. 

#### 2.4.3. Bayesian Multi-Trait Multi-Environment (BMTME) 

Multi-trait and multi-environment prediction were performed with Bayesian Multi-Trait Multi-Environment (BMTME) models, proposed by Montesinos-López et al. [66] which was implemented in the R package BMTME [66]. Briefly, datasets were fitted in the model: Y = X_E_B + Z_1_b_1_ + Z_2_b_2_ + e, where Y is defined as the phenotypic response with a *n* × *L* matrix, n represents observations of a trait and *L* is the number of traits. X_E_ is a design matrix for the environmental effects with an *n* × *I* order, B is defined as beta coefficients with an *I* × *L* matrix. Z_1_ is the design matrix of genotypes with *n* × *J* order, and Z_2_ is the design matrix of genotype x environment interactions with an order of *n* × *JI*. b_1_ is the matrix for genotypic random effects with an order of J × L, followed a distribution as b_1_ ~ *M N* (0, G, Σ_t_), where *M N* represents matrix normal distribution with a mean vector 0 and within and between variance-covariance matrices G and Σ_t_. G is calculated according to Van Raden [67] and assumed known. Σ_t_ is a *L* × *L* matrix for unknown variance -covariance of traits and b_2_ stands for random effects of genotype x environment interactions with a matrix of *JI* × *L* order with an assumed distribution as b_2_ ~ *M N* (0, $\sum_{E}⊗\;G$, Σ_t_). e is the matrix for random residual errors with a *n* × *L* dimension, distributed as *M N* (0, I_n_, R_e_). 

#### 2.4.4. Cross Validations of the Prediction Accuracy (PA)

Prediction accuracies of TP sizes and TP optimizations were obtained by 100 iterations from these defined TP with the genomic selection model rrBLUP [64]. For all other tests, predictions accuracies were estimated from the 5-fold cross validation scheme. With this scheme, lines were randomly divided into 5 subsets and four of the five subsets were combined as the TP, and the remaining fifth fold was treated as the validation set. This scheme was applied to each fifth fold of these five subsets and this process was repeated 10 times. Prediction accuracy was calculated as Pearson’s correlation between the GEBVs obtained from GS models and the observed phenotypic data. 

## 3. Results

### 3.1. Effects of Training Population (TP) Size, TP Optimization and Models of Prediction 

**Figure 1:**
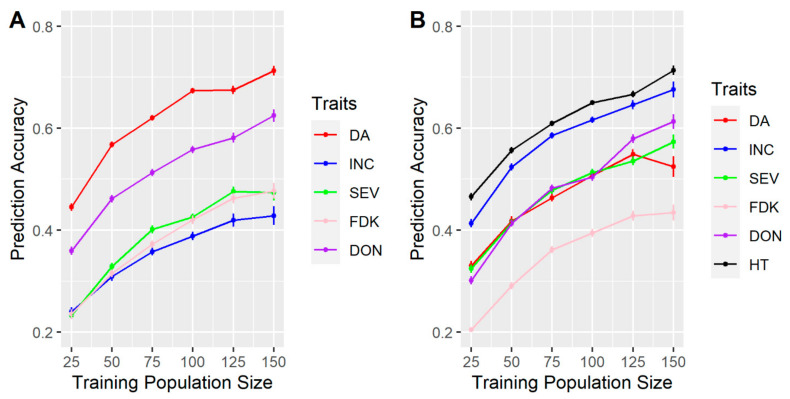
The effect of training population (TP) size on accuracies of genomic selection for fusarium head blight (FHB)-related traits in (**A**) Year 2015; (**B**) Year 2016. Prediction accuracy is the mean value of 100 iterations from the randomly sampling TP of the same size. Days to anthesis (DA), incidence (INC), severity (SEV), Fusarium damaged kernels (FDK), deoxynivalenol (DON) and plant height (HT). Standard error is indicated for each point.

We used the FS population, with data from MB, to assess the effects of the population size on the accuracy of genomics prediction. For all traits, the prediction accuracy considerably increased by increasing the size of the TP from 25 to 150 lines in both year-independent tests (Figure 1). The accuracy did not reach a plateau at the maximum TP size of 150 lines for any of the traits except DA in 2016 (Figure 1B). 

In addition, for all test TP sizes, for DA, a higher accuracy was observed in 2015 (Figure 1A) than in 2016 (Figure 1B). INC and SEV were both observed with higher accuracies in 2016 than 2015 (Figure 1). DON consistently showed higher accuracies across two-year tests, in contrast there was consistently lower accuracies of FDK across years (Figure 1). HT showed the highest accuracy in 2016 compared to all other traits (Figure 1). 

**Figure 2:**
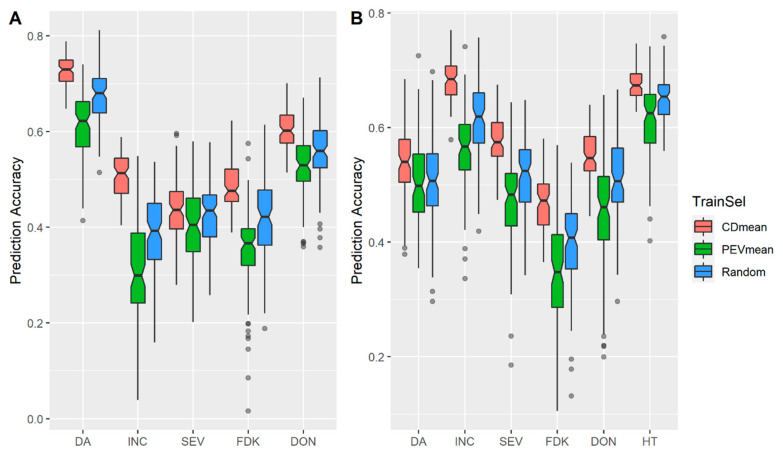
Effects of training population optimization methods on accuracies of fusarium head blight (FHB)-related traits in (**A**) Year 2015; (**B**) Year 2016. Training population (TP) with a size of 100 lines was randomly selected or optimized with CDmean or PEVmean algorithms. Days to anthesis (DA), incidence (INC), severity (SEV), Fusarium damaged kernels (FDK), deoxynivalenol (DON) and plant height (HT). Prediction accuracy is the mean value of 100 iterations for each defined TP.

In addition to the TP size, we also evaluated the effect of TP optimization algorithms with same datasets above. As shown in Figure 2, TP selection using CDmean always performed better than the PEVmean and random sampling approaches with PEVmean showing lower accuracies than random sampling for all traits in both 2015 (Figure 2A) and 2016 (Figure 2B). When the TP size was reduced to 50 lines the same trends were observed, with the best performance from CDmean optimization (Figure S1). 

**Figure 3:**
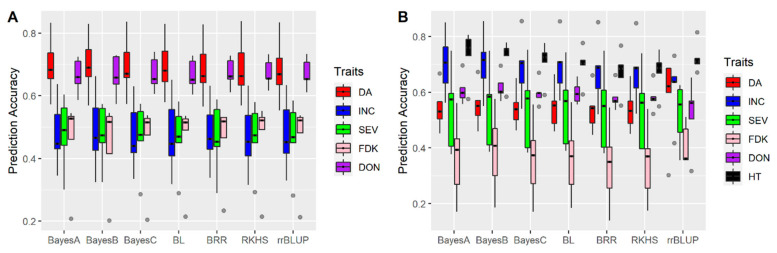
Accuracies of genomic prediction models in (**A**) Year 2015; (**B**) Year 2016. Models include five Bayesian’s approaches: BayesA, BayesB, BayesC, Bayesian least absolute shrinkage and selection operator (BL), Bayesian Ridge Regression (BRR) and two other models: reproducing kernel Hilbert space (RKHS) and ridge regression-best linear unbiased prediction (rr-BLUP). Whiskers represent the upper and lower limit, and the box represents the quartiles Q1 (25%), Q2 (median, thick black line within the box), and Q3 (75%). Each model was evaluated by fivefold cross-validations and repeated 10 times. Days to anthesis (DA), incidence (INC), severity (SEV), Fusarium damaged kernels (FDK), deoxynivalenol (DON) and plant height (HT).

Seven genomic prediction models, including five parametric (rrBLUP, BayesA, BayesB, BayesC, BL, BRR) and one semiparametric approach (RKPHS) were evaluated with the same datasets. In the year 2015, each model had a similar performance for all FHB related traits (Figure 3A). In the year 2016, each model also showed similar accuracy, except rrBLUP showed higher accuracies for DA and SEV, but lower accuracies for DON (Figure 3B). 

### 3.2. Incorporation of Prior Known QTL into the Genomic Prediction Model 

**Figure 4:**
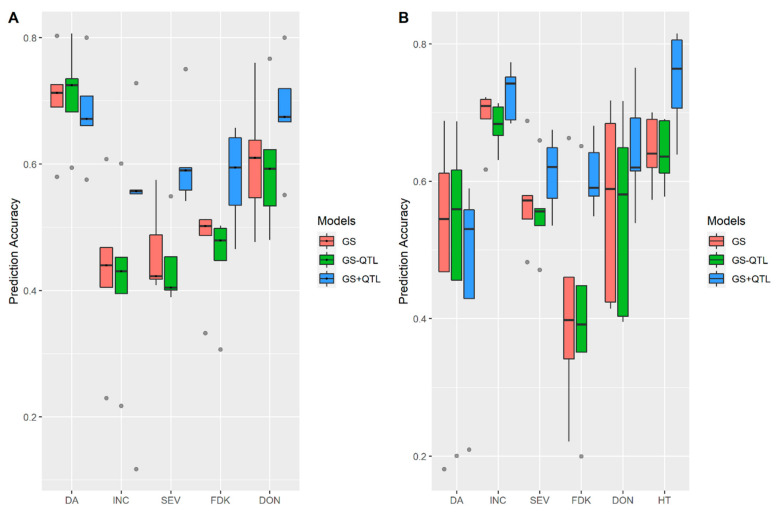
Accuracies of genomic prediction incorporating QTL as fixed effects for FHB related traits in (**A**) Year 2015; (**B**) Year 2016. Results were shown with the genomics selection (GS) model with the whole marker dataset, marker datasets without QTL peak markers (GS−QTL), and GS prediction with QTL as fixed effects (GS+ QTL). Whiskers represent the upper and lower limit, and the box represents the quartiles Q1 (25%), Q2 (median, thick black line within the box), and Q3 (75%). Each model was assessed by fivefold cross-validations and repeated 10 times. Days to anthesis (DA), incidence (INC), severity (SEV), Fusarium damaged kernels (FDK), deoxynivalenol (DON) and plant height (HT).

We evaluated the effect of including QTL as a fixed effect in the genomics prediction model, with the datasets of the FS population from MB. As shown in Figure 4A, in 2015, incorporating QTL into the GS models significantly improved the prediction accuracy for FHB traits including INC, SEV, FDK and DON, but had almost no effect on DA. A similar pattern was also observed in the year 2016 (Figure 4B). We also found in 2016, accuracy of HT prediction was improved by incorporating prior known QTL (Figure 4B). 

### 3.3. Multi-Traits and Multi-Environment Prediction

**Figure 5:**
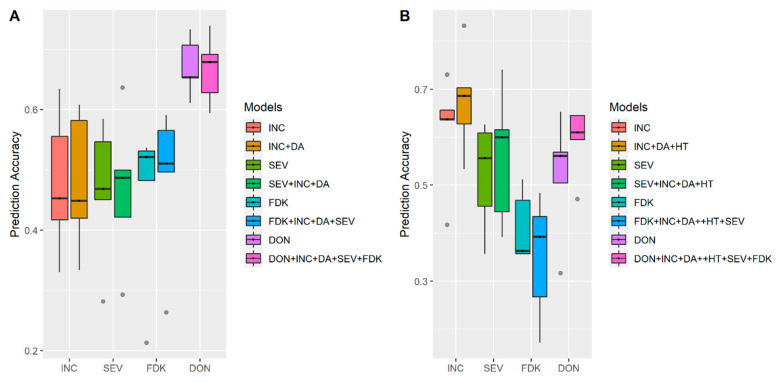
Accuracies of genomic prediction with multi-trait model in (**A**) Year 2015; (**B**) Year 2016. Whiskers represent the upper and lower limit, and the box represents the quartiles Q1 (25%), Q2 (median, thick black line within the box), and Q3 (75%). Each model was assessed by fivefold cross-validations repeated 10 times. Days to anthesis (DA), incidence (INC), severity (SEV), Fusarium damaged kernels (FDK), deoxynivalenol (DON) and plant height (HT). For FHB resistance traits, their corresponding multi-trait models with combination of INC, SEV, FDK, DON, DA and HT were defined as shown by the legend Models.

We first compared the single trait and multi-trait model analysis in the year 2015 with datasets of FS population from MB. As shown in Figure 5A, for FHB related traits, INC, SEV, FDK, and DON, and their corresponding multi-trait models, INC + DA, SEV + INC + DA, FDK + INC + DA + SEV, and DON + INC + DA + SEV + FDK, results of multi-trait models were very similar to the single trait model analysis. For the year 2016, HT was included in the multi-trait model in addition to DA. We found that only for DON, the multi-trait model, DON + INC + DA + HT + SEV + FDK performed better than the single trait model analysis (Figure 5B). 

**Figure 6:**
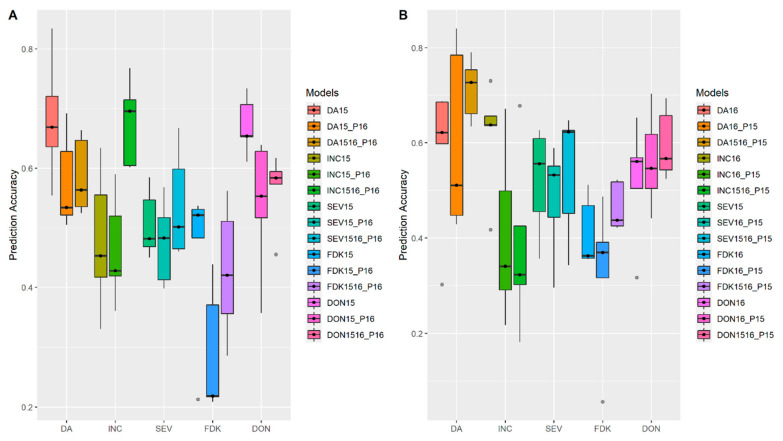
Accuracies of genomic prediction with multi-environment model built from multiple years in the same location and predicted for (**A**) Year 2016; (**B**) Year 2015. Whiskers represent the upper and lower limit, and the box represents the quartiles Q1 (25%), Q2 (median, thick black line within the box), and Q3 (75%). Each model was assessed by fivefold cross-validations repeated 10 times. 15 and 16 represented the year 2015 and 2016. 15_P16 and 16_P15, models were built from 2015 and 2016 and were used to predict performance across the year of 2016 and 2015. 1516_P15 and 1516_P16, models were built from multiple years 2015 and 2016 and were used to predict the year traits performance in Year 2015 and 2016. Days to anthesis (DA), incidence (INC), severity (SEV), Fusarium damaged kernels (FDK), deoxynivalenol (DON) and plant height (HT).

**Figure 7:**
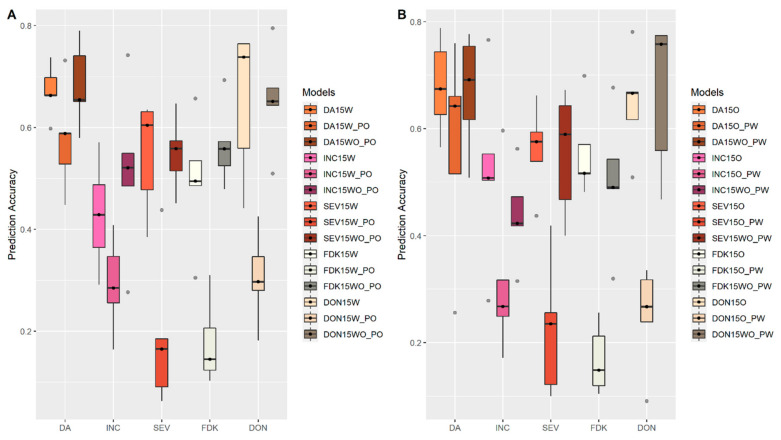
Accuracies of genomic prediction with multi-environment models built from multiple locations to predict performance across locations in the year 2015 (**A**) Prediction of traits for Ottawa, Ontario (O); (**B**) Prediction of traits for Carman, MB (W). Whiskers represent the upper and lower limit, and the box represents the quartiles Q1 (25%), Q2 (median, thick black line within the box), and Q3 (75%). Each model was assessed by fivefold cross-validations repeated 10 times. 15 represented the year 2015. W and O, represented Carman, MB and Ottawa, Ontario. Models built from single environment (SE) at Carman, MB (W), and multiple environment model with two locations, Carman, MB and Ottawa, Ontario (WO) were used to predict traits performance at Ottawa, Ontario (W_PO and WO_PO) and compared to the single environment model at local environment (W), as shown in (**A**). Models built from single environment (SE) at Ottawa, Ontario (O), and multiple environment model with two locations, Carman, MB and Ottawa, Ontario (WO) were used to predict traits performance at Carman, MB (O_PW and WO_PW) and compared to the single environment model’s prediction at local environment (O), as shown in (**B**).

To evaluate the accuracy of the multi-environment prediction model, we adopted a scenario to simplify the complexity of GEI. We first assessed a scenario of prediction accuracy in the same location but across different years with datasets of the FS population from MB. For all FHB related traits, a single environment model built from a one-year trial always had lower accuracy when applied to the other year (Figure 6A,B). When a multi-environment model built from multiple years was applied, it achieved better performance for the prediction across years compared to a single environment model (Figure 6). We then assessed the multi-environment model from the same year but across different locations with datasets of the FS population from both MB and ON. As shown in Figure 7A, models built from the trials at Winnipeg, MB (Western Canada) showed lower accuracy to predict the performance of FHB related traits at Ottawa, ON (Eastern Canada) in the year 2015. A similar pattern was observed when an Eastern Canada model was used to predict the performance in Western Canada (Figure 7B). When a multi-environment model developed from both locations was applied, it always displayed higher prediction accuracy across different locations in 2015 (Figure 7). A similar pattern was observed in 2016 (Figure S2), validating the better performance of the multi-environment model. 

### 3.4. Prediction within and across Populations

The multi-parental population including FS, FM and FE was evaluated for FHB resistance with datasets from MB. This multi-parental population was designed as a nested association mapping (NAM) approach with the FHB resistance line FL62R1, as common parental line. Principal component analysis (PCA) showed that lines from each individual population were grouped together (Figure S3). PC1 differentiated the FE population from both the FS and FM population (Figure S3). Dendrogram of hierarchical cluster analysis on the genomic relationship of this multiple parental population is also consistent with the PCA analysis (Figures S3 and S4), showing that the FS and FM population have higher relatedness to each other, and lower relatedness to the FE population (Figures S3 and S4). 

**Figure 8:**
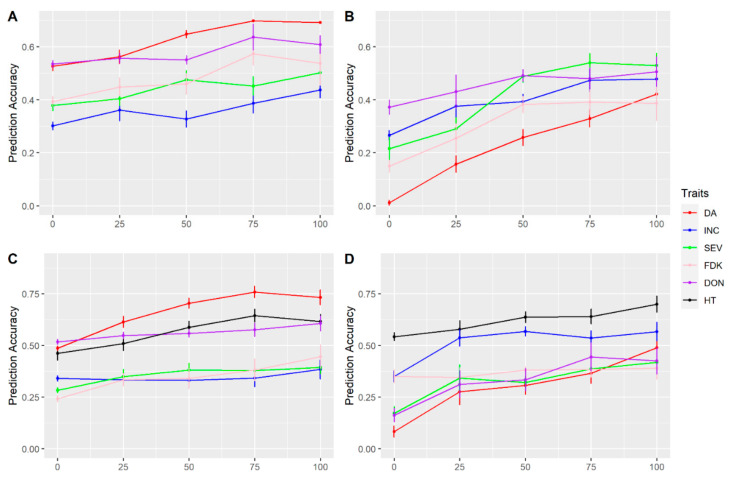
Accuracies of genomic prediction within and across populations. Stetter (FS) population was set as reference population and was used to predict performance on Muchmore population (FM) in (**A**) Year 2015 and Emerson population (FE); (**B**) in Year 2016 and (**C**) FM in Year 2016 and (**D**) FE in Year 2016. Days to anthesis (DA), incidence (INC), severity (SEV), Fusarium damaged kernels (FDK), deoxynivalenol (DON) and plant height (HT). Prediction accuracy is the mean value by fivefold cross-validations repeated 10 times. 0–100% presented lines of tested populations in the TP, increased from 0 to 100 percent (and reference population decreased from 100 to 0%). Standard error is indicated for each point.

To evaluate predictabilities across populations, we set the FS population as the reference population and used it to predict performance on the other two test populations. As shown in Figure 8, when TP composition changed from 0 (all lines were from FS population) to 100% (all lines were from test populations), accuracies were consistently increased for all FHB related traits in both two-year tests. When the TP was at 50% of reference population, lines from the test population can get a relatively reasonable accuracy, varying from ~0.3 to 0.65 for these different traits (Figure 8). When the reference FS population was used to predict the FM (Figure 8A,C) and FE population (Figure 8B,D), higher accuracies were observed for the FM population.

## 4. Discussion

### 4.1. TP Size 

The gradual increase in accuracies for FHB related traits along with the increased TP size agrees with many previous research, including FHB resistance in wheat [30,58] and barley [28], agronomics traits in winter wheat [68] and maize [69]. The size of the training set has a big impact on the accuracy of GS, and a TP of sufficient size is required for good prediction [33,40,48]. However, there are practical reasons the size is constrained (i.e., additional costs with the larger field tests) and there is a TP size where the gains are diminished [28,43]. In barley, it was reported that increasing the size of TP beyond 200 lines has little effect on the accuracy of the FHB resistance prediction [28]. Arruda et al. [30] found that with a TP size of 192 lines, all FHB related traits in wheat except index reaching the plateau. Isidro et al. [41] evaluated five agronomic traits in wheat and found that accuracy prediction plateaued with a TP size at 300 lines. We observed that the prediction accuracy was still increasing when the TP size reached the maximum of our test, 150 lines. Considering that FHB resistance is a complex trait that is controlled by many minor genes that are significantly affected by GEI and with relatively lower phenotyping accuracy, a TP with a size around 200 lines, or more will be needed to achieve better accuracy for FHB resistance in wheat. 

### 4.2. Design TP with Optimization Algorithms

Larger training population size tends to increase the accuracy of the genomic selection, however, a reasonable accuracy can be achieved with a small TP size, given an appropriate design [44,45,46,70]. In addition, knowing how well the model predicts could allow breeders to better allocate resources to apply the GS in the breeding program [41]. Thus, designing an optimal TP is central to the success of the GS in the breeding program. CDmean and PEVmean were originally applied to optimize the TP by Rincent et al. [46] and later these approaches were advanced to stratified CDmean (StratCDmean) and Selection of Training Populations by Genetic Algorithm (STPGA) [41,44,45]. In present study, we found that CDmean consistently outperformed PEVmean for all FHB related traits and at a different TP size. The better performance of CDmean to design an optimal TP with higher accuracy agrees with previous findings for complex traits from many other crops [41,46,47,71]. Together, all of these findings indicated that CDmean may be a promising approach to optimize the TP for better accuracies. Tiede and Smith [71] reported that all optimization algorithms performed better than random sampling TP. However, in our study, PEVmean had consistently worse accuracies compared to random sampling in different years and for all evaluated FHB related traits. Isidro et al. [41] also found that PEVmean showed lower accuracies for florets per panicle and flowering time in wheat with an intermediate TP size, a size comparable to the TP size of our study (around 100–150 lines). There is a need to further explore the cause of this lower accuracy of PEVmean. In addition, most current optimization algorithms only use the genomics information of the TP, except the STPGA, which also incorporated the information from the testing population [44]. In the future, new advanced optimization algorithms that can design the TP with a better predictability will pave the way for the design of the TP, and thus can broadly benefit the GS application in the breeding program. 

### 4.3. Models of Prediction 

There are a large number of models that can be used to predict phenotypic traits and these methods can be simply classified into parametric and non-parametric (or semi-parametric) approaches [72]. These methods varied in their accuracies for different traits as parameters varied according to different assumptions to appropriately suit the genetic architecture underlying that targeted trait [48,49,50]. The majority of models used for genomic prediction are Bayesian based models and rrBLUP (or GBLUP, equal to rrBLUP) model [51,64]. In the present study, we found seven models including both parametric and non-parametric approaches had very similar performance for all FHB related traits across different years. Heffner et al. [43] also observed no difference for different GS models, tested on thirteen agronomic traits in wheat. With their findings as ours, GS models may play a minor effect on the accuracy to predict FHB resistance and agronomics traits in wheat.

### 4.4. Incorporation of Prior Known QTL

Previous studies with both simulated and empirical datasets in different crops, including rice, maize, sorghum and wheat demonstrated that incorporating large effect QTL as fixed effects into GS can lead to better predictability [54,55,58,73,74,75,76]. In our study, we also found that including QTL into GS model as fixed effects can considerably improve accuracies for FHB resistance traits including INC, SEV, FDK and DON. This benefit was also validated by the test in different environments. Arruda et al. [74] also observed that including QTL as fixed effects can significantly improve accuracies of GS for INC, SEV, FDK, but with no effect on DON. Beside FHB traits, Herter et al. [58] 2019 found higher accuracies for the heading date and plant height with QTL as fixed effects in the GS model, while our study found including fixed effect QTL influenced the prediction accuracy of plant height but had little or no effect on heading date. A simulation study [54] demonstrated that including known major genes as fixed effects can increase prediction accuracy when a substantial amount of phenotypic variance can be explained by major genes. Our previous study [17] identified that a major QTL from FL62R1 on chromosome 4B contributed to the FHB resistance and plant height. In addition, FHB1 was found as the second largest QTL from FL62R1 that conferred FHB resistance [17]. However, only quite minor QTL were identified for the heading date. Thus, our findings were in agreement with results from Bernardo [54]. Recently, Larkin et al. [57] reported that GS plus QTL performed worse than native GS, while Rice and Lipka [76] showed that the benefits of GS plus QTL were on a trait-by-trait basis. Therefore, application of the GS plus QTL model should take into account the trait, effect of QTL, and the property of the genetic architecture underlying the trait. An additional complication is that candidate genes controlling many traits are still not yet cloned, and most times, markers associated with the trait were used instead of functional genes. This may affect the accuracy of the GS plus QTL model. Indeed, a negative impact on the GS was noted by Spindel et al. [55] when inaccurate or inappropriate fixed effects were incorporated into the GS model. 

### 4.5. Predictability with Multi-Trait and Multi-Environment Models

Many studies, including our previous study on the FHB resistance of FL62R1 identified that flowering time and plant height are associated with FHB resistance [17,21,23,25]. In addition, different FHB resistance components, including INC, SEV, FDK and DON may partially share the same genetic architecture [17,21,25]. Jia and Jannink [52] reported that for genetically correlated quantitative traits, multivariate genomic selection (MVGS) model on multiple correlated traits outperformed the single-trait (univariate) GS model. With the application of the MVGS model on FHB related traits including INC, SEV, FDK and DON and plant height, Larkin et al. [57] found that MVGS performed better for all these FHB resistance traits except for DON. In our study, we found MVGS did not perform better than the single trait prediction model for all these FHB related traits in the test of Year 2015. This finding was supported by the other independent test in the year of 2016, except for DON, where MVGS had better accuracy than the single trait model. Previous study in wheat quality and yield traits, showed that MVGS models did not perform better than the single trait model except yield [77,78]. These discrepancies on accuracies of MVGS models may be partially caused by different genetic architecture, mechanism and interactions of genes underlying genetically correlated traits. 

In contrast, when we applied multi-environment (MET) models across different years or locations, MET models that incorporated GEI always performed better than single environment models for all FHB related traits and for DA and HT. Lado et al. [79] performed MET model analysis with 35 location–year combinations and found that higher predictive ability can be achieved by modeling GEI in the GS models. Lado et al. [79] also found modeling GEI for GS quite complicated, and that borrowing information from relatives evaluated in multiple environments and modeling them is important for MET prediction accuracies. Our findings indicated that designing a TP with phenotypic traits generated from environments similar to environments of the test population may be crucial for the success of MET prediction in the breeding program. 

### 4.6. Predictability within and across Populations 

The relatedness between the TP and tested population is critical for the prediction of complex traits [36]. With FS as reference population, we found that changing the TP compositions from 0% of the tested population to 100% (FS decreased from 100% to 0%), increased accuracies for all FHB related traits across different years. This was supported by findings in maize [80] and durum wheat [81], which all indicated that relatedness between TP and test population should be the key consideration for GS application. In addition, we found when FS was used as TP, it better predicted the more closely related FM population than the more distantly related FE population. Parental line Stettler from FS and Muchmore from FM populations are two Canadian western red spring wheat varieties that were developed from the AAFC Swift-Current breeding program, and share the same parent, Superb, in their pedigrees [60,61]. The FE population was generated from Emerson, a winter wheat that is the only R rated FHB resistance wheat in Canada [62]. This pedigree information validated the higher genetic relatedness between the FS and FM population than the FE population. It should be noted that the FS TP and the test populations all share the major 4B QTL for FHB resistance that derives from the common FHB resistant parental line FL62R1 [17]. Thus, findings from this study may have limits when attempting to apply to broad applications. 

## 5. Conclusions

In this study, with multiple connected bi-parental populations, we evaluated factors that can affect accuracies for the prediction of FHB related traits in wheat. We found a TP size around 200 lines, or more, is needed to achieve good accuracies for FHB resistance. Incorporating prior known QTL information of these traits can significantly improve predictabilities. Multi-environment modelling and including phenotypic data from the TP with relevance to the test population led to considerably better predictabilities across environments. Relatedness between TP and testing population is the key for accuracies, but accurately applying GS across less related populations remains a challenge.

## 6. Patents

Not Applicable.

## Data Availability

All data generated during this study are included in this published article (and its Supplementary Information files).

## Supplementary Materials

The following are available online at https://www.mdpi.com/article/10.3390/biology10080756/s1, Figure S1: Effects of training population (TP) optimization on the accuracy of fusarium head blight (FHB)-related traits in (A) Year 2015; (B) Year 2016. A TP with a size of 50 lines was randomly selected or optimized with CDmean and PEVmean. Prediction accuracy is the mean value of 100 iterations for each of these defined TP. Days to anthesis (DA), incidence (INC), severity (SEV), Fusarium damaged kernels (FDK), deoxynivalenol (DON) and plant height (HT), Figure S2: Accuracies of genomic prediction with multi-environment models built from multiple locations predicted performance across locations in the year 2016 (A) Prediction of traits for Ottawa, Ontario (O); (B) Prediction of traits for Carman, MB (W). Whiskers represent the upper and lower limit, and the box represents the quartiles Q1 (25%), Q2 (median, thick black line within the box), and Q3 (75%). Each model was assessed by fivefold cross-validations repeated 10 times. 16 represented the year 2016. W and O, represented Carman, MB and Ottawa, Ontario. Models built from single environment (SE) at Carman, MB (W), and multiple environment model with two locations, Carman, MB and Ottawa, Ontario (WO) were used to predict traits performance at Ottawa, Ontario (W_PO and WO_PO) and compared to the single environment model at local environment (W), as shown in (A). Models built from single environment (SE) at Ottawa, Ontario (O), and multiple environment model with two locations, Carman, MB and Ottawa, Ontario (WO) were used to predict traits performance at Carma, MB (O_PW and WO_PW) and compared to the single environment model’s prediction at local environment (O), as shown in (B), Figure S3: Principal component analysis (PCA) of the population structure of the multi-parental population. Blue dots represented Emerson population (FE), red and green were Stettler population (FS) and Muchmore population (FE). X-axis and Y-axis represented variations explained by PC1 and PC2, Figure S4: Dendrogram of hierarchical cluster analysis heatmap of the genomic relationship matrix for the wheat multi-parental population. Blue branches represented Emerson population (FE), red and green branches were Stettler population (FS) and Muchmore population (FE).
