# Supplementary figures and images for: Evaluation of Genomic Prediction for Fusarium Head Blight Resistance with a Multi-Parental Population
Source: Biology (Basel). 2021 Aug 6;10(8):756. doi: 10.3390/biology10080756 (PMC8389552; doi:10.3390/biology10080756)

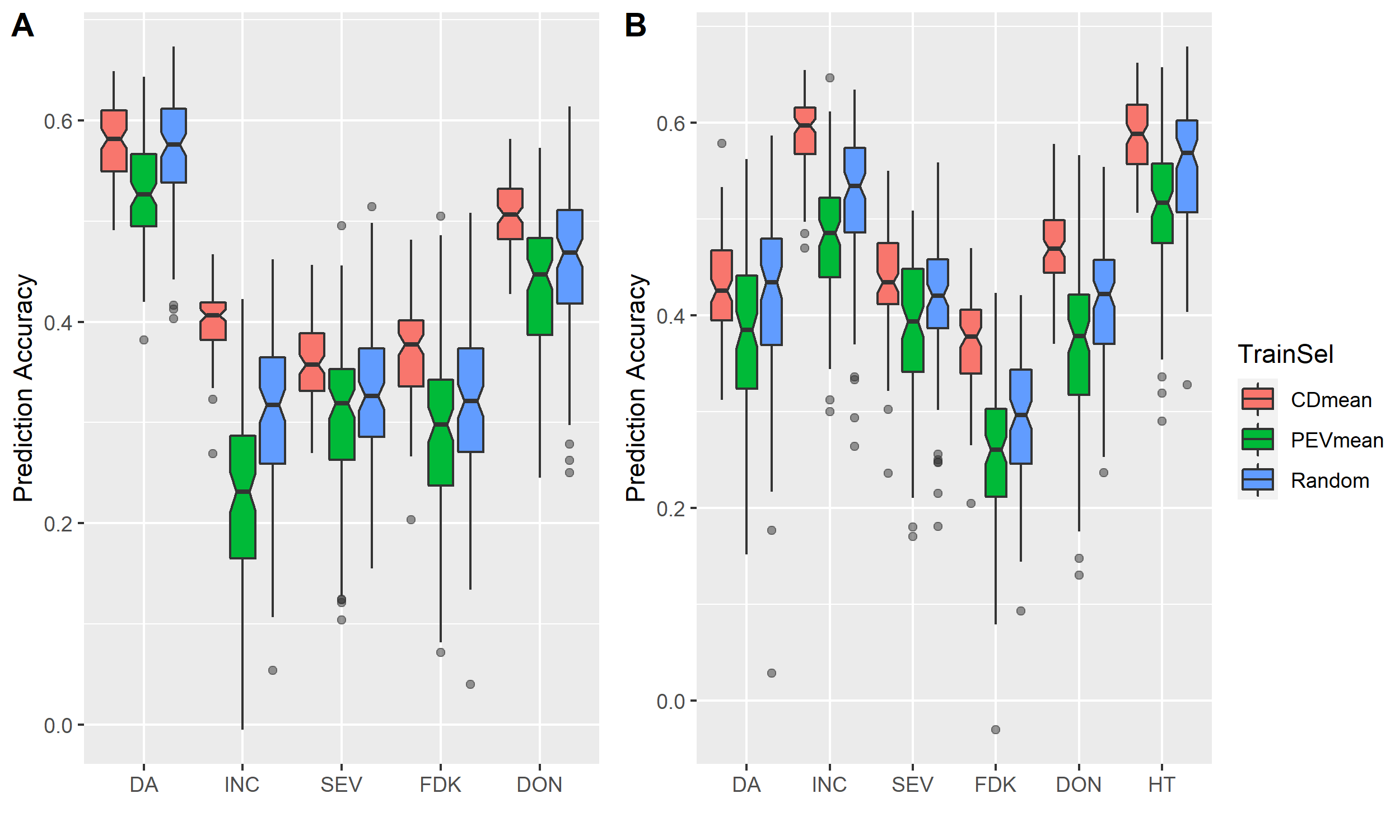

Supplement: Supplementary file 1 [file biology-10-00756-s001.zip › Figure S1..tif]

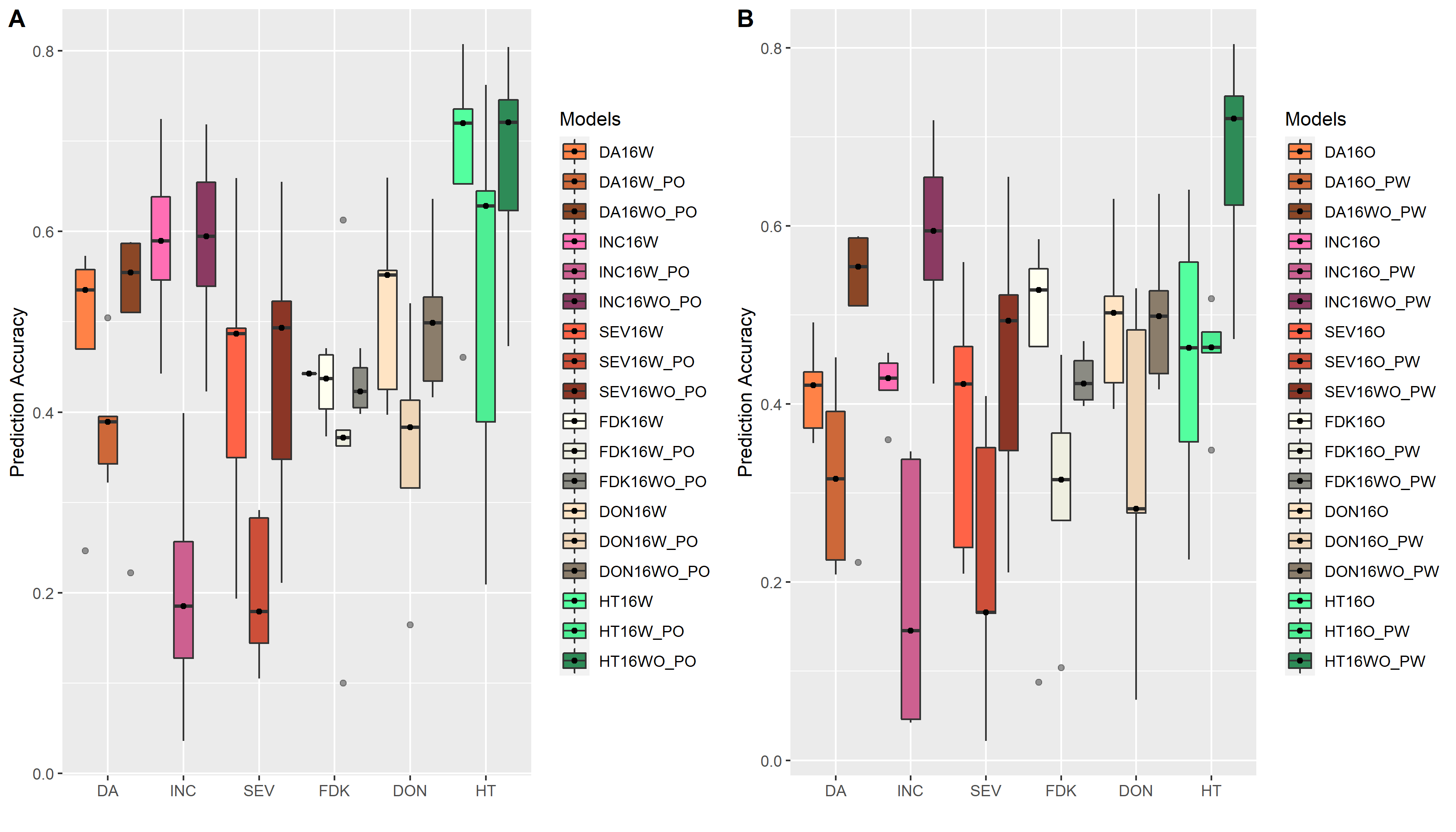

Supplement: Supplementary file 1 [file biology-10-00756-s001.zip › Figure S2..tif]

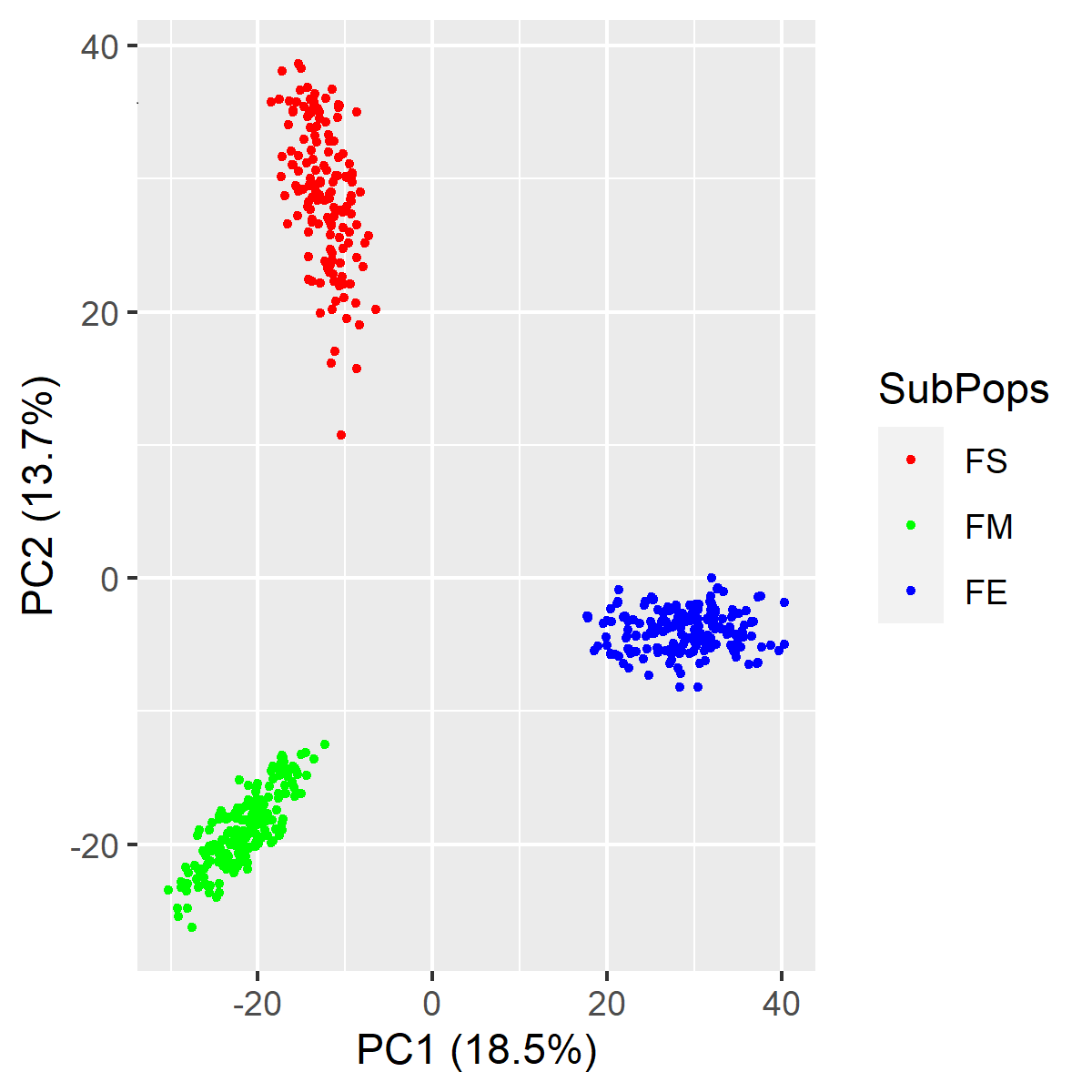

Supplement: Supplementary file 1 [file biology-10-00756-s001.zip › Figure S3..tif]

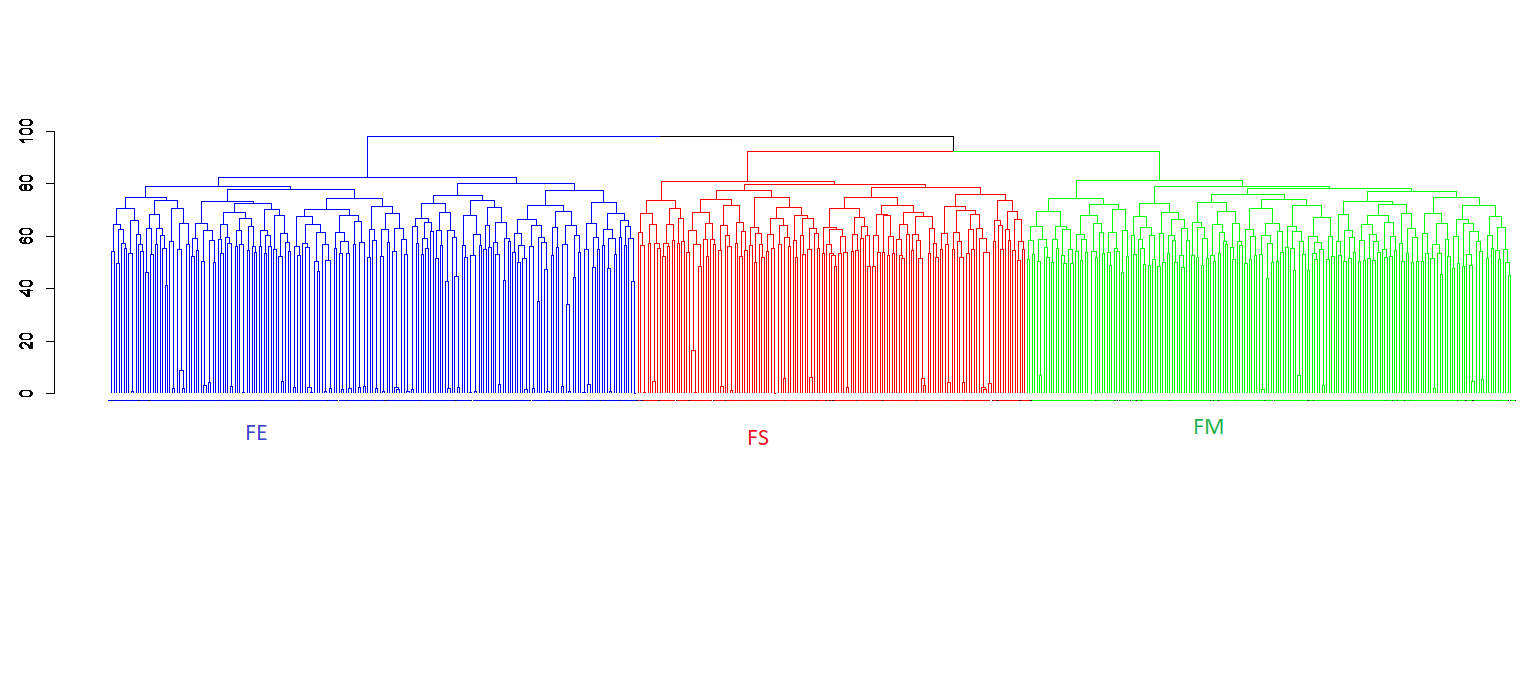

Supplement: Supplementary file 1 [file biology-10-00756-s001.zip › Figure S4..tif]
